# S249. IS INTERNET HARMFUL FOR PSYCHOTIC PATIENTS?

**DOI:** 10.1093/schbul/sby018.1036

**Published:** 2018-04-01

**Authors:** Lucia Bonet, Blanca Llacer, Miguel Hernandez, David Arce, Ignacio Blanquer, Carlos Cañete, Maria Jose Escarti, Julio Sanjuan

**Affiliations:** 1 Universidad de Valencia; 2 CIBERSAM; 3 Clinic Hospital Valencia, CIBERSAM; 4 Universidad Politecnica de Valencia; 5 Peset Hospital

## Abstract

**Background:**

Developments in electronic health (e-Health) interventions for psychotic patients have been possible since the growing access and use of internet and electronic devices in past 10 years (Bonet et al. 2017). However, before proceeding further on develop these interventions; limited knowledge exists about the impact of internet and new technologies on the mental health of these psychotic patients. The aim of this study is to assess the benefits and risks of new technologies usage in a survey of patients diagnosed with psychotic disorders. We analyzed the relationship between experiences and opinions about internet and demographic and clinical characteristics of the sample and patterns of use of these technologies.

**Methods:**

Structured questionnaire was designed. This questionnaire was divided in three parts: 1) clinical and demographic information, 2) access and use of technologies, and 3) experiences and opinions about internet. In total, 97 patients diagnosed with psychotic disorder participated in this cross-sectional study. Mean age of the sample was 37.06 (SD=12.9), 72.2% of participants were male, 84.5% were single and 60.8% had achieved secondary education. Main diagnoses in the sample were First Episode of Psychosis (45.4%) and Schizophrenia (34%) and 64.9% of patients had a length of illness lower than 72 months

**Results:**

The percentage of patients who daily acceded to internet was 63.9% while 21.6% weekly acceded. 90.7% of participants owned a mobile phone and 68% had a social media account. Related to feelings about internet, 60.8% of patients felt socially linked due to internet usage and 78.4% felt informed. However, 22.7% felt frustrated and 19.6% felt suspicious. Internet was considered as a benefit for mental health for 46.4% of patients, while 38.1% have had unpleasant experiences related to its usage, 24.7% have had internet-related relapses and 26.8% expended excessive time online. Significant association was found between feeling informed and frequency of access to internet (χ2= 6.17 p=0.05), however any other significant association was found between feelings about internet and clinical or demographic characteristics or patterns of use of technology. According to experiences, significant associations were found between internet-related relapses and length of illness (χ2= 4.74 p=0.03), frequency of internet access (χ2= 9.76 p<0.01) and social media ownership (χ2= 5.55 p=0.02). Expending excessive time on internet was found significant associated to age of the sample (χ2= 6.57 p=0.04), employment status (χ2= 10.73 p=0.03), frequency of access to internet (χ2= 10.15 p<0.01) and social media ownership (χ2= 9.62 p<0.01). Association between stop taking medication because of information read on the internet and level of education was also found (χ2= 9.03 p=0.01).

**Discussion:**

Despite the general positive feelings about internet usage, percentages between 38-19% of patients had a negative vision of internet. Furthermore, frequency of access to internet and social media ownership have been found associated to internet-related relapses and potential pathological use of internet (excessive time on it). Younger patients, recent diagnosis of psychosis and being in a non-active employment situation seem to be related to these pathological results too. To our knowledge, this is the first study to describe the potential risks about internet usage in patients diagnosed with psychotic disorders, however further studies are needed.

Reference:

1. Bonet L, et al Use of mobile technologies in patients with psychosis: A systematic review. Rev Psiquiatr Salud Ment. 2017; 10 (3): 168–178

